# Generation of Foxp3^+^CD25^−^ Regulatory T-Cell Precursors Requires c-Rel and IκB_NS_

**DOI:** 10.3389/fimmu.2019.01583

**Published:** 2019-07-10

**Authors:** Marc Schuster, Carlos Plaza-Sirvent, Alexander Visekruna, Jochen Huehn, Ingo Schmitz

**Affiliations:** ^1^Systems-Oriented Immunology and Inflammation Research Group, Department of Experimental Immunology, Helmholtz Centre for Infection Research, Braunschweig, Germany; ^2^Medical Faculty, Institute for Molecular and Clinical Immunology, Otto-von-Guericke University, Magdeburg, Germany; ^3^Institute for Medical Microbiology and Hygiene, Biomedical Research Center (BMFZ), Philipps University of Marburg, Marburg, Germany; ^4^Department of Experimental Immunology, Helmholtz Centre for Infection Research, Braunschweig, Germany

**Keywords:** cell differentiation, common γ-chain cytokines, NF-κB, regulatory T cell, thymus, transcription factor

## Abstract

Next to the classical developmental route, in which first CD25 and subsequently Foxp3 are induced to generate thymic regulatory T (Treg) cells, an alternative route has been described. This alternative route is characterized by reciprocal induction of Foxp3 and CD25, with CD25 induction being required to rescue developing Treg cells from Foxp3-induced apoptosis. NF-κB has been demonstrated to be crucial for the development of thymic Treg cells via the classical route. However, its impact on the alternative route is poorly characterized. Using single and double deficient mice for key regulators of the classical route, c-Rel and IκB_NS_, we here demonstrate that NF-κB is essential for the generation of alternative CD25^−^Foxp3^+^ precursors, as well. Thus, c-Rel and IκB_NS_ govern both routes of thymic Treg cell development.

## Introduction

Regulatory T (Treg) cells constitute a subset of CD4^+^ T cells, which is characterized by the expression of the winged-helix transcription factor forkhead box P3 (Foxp3) (1). Treg cells are an essential part of peripheral tolerance (2) and the regulation of Treg cell generation is essential to maintain immune homeostasis (3).

Treg cell development depends on TCR and common gamma chain (γ_c_) cytokine-mediated signals (4, 5). A prominent two-step model suggests that a CD25^+^Foxp3^−^ cell population contains Treg cell precursors, which develop upon TCR stimulation of CD4 single-positive (SP) thymocytes (6). This stimulation causes the induction of CD25, which makes these CD25^+^Foxp3^−^ Treg cell precursors receptive for γ_c_-mediated stimuli via e.g., IL-2 or IL-15, which in a second step lead to the differentiation of CD25^+^Foxp3^−^ precursors into CD25^+^Foxp3^+^ mature Treg cells (6, 7). Next to this classical route, an alternative developmental route has been suggested, in which mature CD25^+^Foxp3^+^ Treg cells arise from CD25^−^Foxp3^+^ cells (8, 9). These alternative precursor cells require stimulation via γ_c_ cytokines to upregulate CD25 and to prevent Foxp3-induced apoptosis, hence, allowing differentiation into mature Treg cells in a reversed fashion regarding the order of CD25 and Foxp3 induction (8–11).

On the molecular level, current models suggest that NF-κB is a pioneer transcription factor (12) controlling Foxp3 induction during Treg cell development. Especially two NF-κB components, c-Rel and IκB_NS_ (encoded by the *Nfkbid* gene), are crucial for directly activating *Foxp3* expression (13–17). Specifically, c-Rel and IκB_NS_ bind to the core promoter and the conserved non-coding sequence (CNS) 3 of the *Foxp3* locus (16, 17), suggesting that c-Rel and IκB_NS_ act in a common complex and cooperatively regulate Treg cell generation. In addition, NF-κB also promotes another, independent step of Treg cell maturation, namely CD25 induction (18, 19). We could recently show that CD25^−^Foxp3^−^ cells expressing CD122, which constitutes the IL-2 receptor β-chain, may give rise to CD25^+^Foxp3^−^ Treg cell precursors of the classical developmental route and can, thus, be considered as Treg cell pre-precursors (19).

Although several studies indicated an important role of NF-κB components, like c-Rel, for the generation of classical CD25^+^Foxp3^−^ Treg cell precursors, the role of NF-κB for the generation of CD25^−^Foxp3^+^ alternative Treg cell precursors is not well-defined. Moreover, it remains unknown how responsiveness of alternative Treg cell precursors to IL-2 is achieved, since they do not express CD25 (the IL-2 receptor α-chain). In this study, we report that the generation of recently described alternative CD25^−^Foxp3^+^ Treg cell precursors required IκB_NS_ and c-Rel, since alternative precursor cells were reduced to a similar extent in c-Rel-deficient, IκB_NS_-deficient and double knock-out (DKO) mice. Corresponding to the recently proposed Treg cell pre-precursors of the classical route, CD25^−^Foxp3^+^ alternative Treg cell precursors expressed similar amounts of CD122, and c-Rel contributes to their transition into Foxp3^+^CD25^+^ mature Treg cells.

## Method

### Mice

IκB_NS_-deficient (*Nfkbid*^−/−^) mice (17, 20), c-Rel-deficient (*Rel*^−/−^) (21, 22), c-Rel/IκB_NS_ (DKO) double-deficient (19) and Foxp3^DTR−eGFP^ (DEREG) mice (23) used for this study were described before. All animal procedures were performed at the animal facility of the Helmholtz Centre for Infection Research (Braunschweig, Germany) under specific pathogen free conditions and according to current European law with written consent of local authorities.

### Flow Cytometry

For flow cytometry, cells were isolated from the respective organs of mice. 1 * 10^6^ cells were washed twice with PBS in small flow cytometric tubes. Cells were incubated for 30 min with fixable Live/Dead 1:1000/PBS at 4°C. Afterwards cells were washed with PBS and incubated for 15 min with antibodies in FACS-buffer (2% BSA/PBS) and afterwards washed with PBS. For CD122 staining cells were additionally incubated with streptavidin-PE. All antibodies [CD4-PacificBlue (Biolegend 100531), CD8-APC (Biolegend 100712), CD8-PerCPeFluor710 (Biolegend 100734), CD25-APC (Biolegend 102012), CD25-FITC (Biolegend 102006), GITR-PECy7, CD122-biotin (Biolegend 123206)] and Streptavidin-PE (eBioscience 12-4317-87) were used in 1:1000 dilution, except anti-humanCD2-FITC (Biolegend 300206) and anti-Foxp3-PE (eBioscience 12-5773-80) antibodies, which were both used in 1:200 dilution.

### *In vitro* Treg Cell Maturation Assay

Thymocytes from mice with the indicated genotype were stained with anti-CD4-PacificBlue (Biolegend 100531) and anti-CD8-APC (Biolegend 100712) to sort CD4^+^CD8^−^ CD25^−^ eGFP^+^ (Foxp3) Treg precursors via fluorescence-activated cell sorting (FACS Aria II; BD Biosciences). In DEREG mice, eGFP reports Foxp3 expression and allows for purification of viable Foxp3^+^ cells. 15,000 cells per well were directly sorted into a 96-well round bottom plate. Subsequently, cells were stimulated with 100 ng/ml IL-15 for 24 h or left untreated. The generation of Treg cell precursors and mature Treg cells was determined via CD25 and Foxp3 staining and flow cytometry.

### Statistics

The Graph Pad Prism Software (GraphPad Software) was used for all statistical analyses. To determine statistical significance, the one-tailed or two-tailed Mann–Whitney *U*-test or ANOVA tests were used and error bars represent the standard error of the mean (SEM).

## Results

### Alternative CD25^−^Foxp3^+^ Treg Cell Precursor Generation Depends on NF-κB

**Figure 1:**
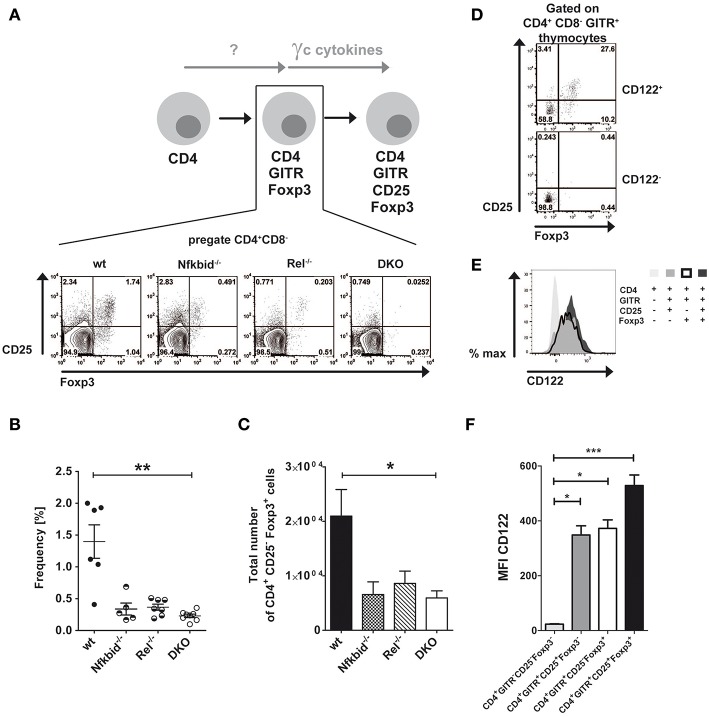
Foxp3^+^CD25^−^ Treg cell development depends on NF-κB and CD122 expression. **(A)** Scheme of Treg cell development via the alternative Foxp3^+^CD25^−^ Treg cell precursor route and corresponding dot blots of wildtype, IκB_NS_-deficient (*Nfkbid*^−/−^), c-Rel-deficient (*Rel*^−/−^) and double deficient (DKO) mice. The indicated Foxp3^+^CD25^−^ Treg cell precursors are the population in the lower right gate of the representative dot blots. **(B)** Frequencies of Foxp3^+^CD25^−^ Treg cell precursors are depicted from wildtype (*n* = 6 mice), IκB_NS_-deficient (*Nfkbid*^−/−^; *n* = 5 mice), c-Rel-deficient (*Rel*^−/−^; *n* = 7 mice) and DKO (*n* = 8 mice) mice. Data is pooled from 5 independent experiments. **(C)** Total numbers of Foxp3^+^CD25^−^ Treg cell precursors from wildtype (*n* = 8 mice), IκB_NS_-deficient (*Nfkbid*^−/−^; *n* = 7 mice), c-Rel-deficient (*Rel*^−/−^; *n* = 6 mice) and DKO (*n* = 8 mice) mice. **(D)** Analysis of Foxp3 and CD25 expression in the CD122^+^ and CD122^−^ populations. The indicated alternative Foxp3^+^CD25^−^ Treg cell precursors are in the lower right gate. **(E)** Histogram overlay of CD122 expression in alternative Foxp3^+^CD25^−^ (bold line), classical Foxp3^−^CD25^+^ Treg cell precursors (dark gray), Foxp3^+^ CD25^+^ Treg cells (black) and CD4^+^ single positive thymocytes (light gray). **(F)** Statistical summary of CD122 MFI of (E) (*n* = 9, each). Statistics (B, C and F) performed via Kruskal-Wallis (ANOVA) test and Dunns post-test. *p < 0.05, **p < 0.01, ***p < 0.001, not indicated = not significant.

Thymic Treg cells develop from CD4SP thymocytes by inducing CD25 and Foxp3 expression in a two-step manner depending on TCR-induced and **γ**_c_ cytokine-mediated signals (6, 7). Next to this classical route, an alternative route was proposed for the thymic development of Treg cells, in which Foxp3 and CD25 induction is reversed (8). We wondered, whether NF-κB signaling contributed to the generation of alternative CD25^−^Foxp3^+^ Treg cell precursors similarly to what has been described for classical CD25^+^Foxp3^−^ Treg cell precursors (17–19, 24). To this end, we analyzed CD25 and Foxp3 expression in CD4^+^GITR^+^ thymocytes from wildtype, IκB_NS_-deficient, c-Rel-deficient and DKO mice (Figure 1A). Our analyses revealed reduced frequencies (Figure 1B) and absolute numbers (Figure 1C) of alternative Treg cell precursors in IκB_NS_-deficient, c-Rel-deficient and DKO mice. Since the reduction of CD25^−^Foxp3^+^ Treg cell precursors was similar in these mice, c-Rel and IκB_NS_ appear to contribute to the alternative route of Treg cell development in a common pathway. Alternative CD25^−^Foxp3^+^ Treg cell precursors express CD122.

Interestingly, γ_c_ signaling induces the differentiation of alternative CD25^−^Foxp3^+^ Treg cell precursors into Foxp3^+^CD25^+^ mature Treg cells (8). Since we described recently that only CD122^+^ T cells give rise to CD25^+^Foxp3^−^ Treg cell precursors in the classical route of Treg cell development (19), we here analyzed whether CD25^−^Foxp3^+^ alternative Treg cell precursors do express CD122 as well. Indeed, we detected CD25^−^Foxp3^+^ cells exclusively in the compartment of CD122^+^ CD4SP thymocytes, but not within the CD122^−^ fraction (Figure 1D), indicating a comparable role for CD122 in both Treg cell developmental routes. Furthermore, we analyzed CD122 expression during Treg cell development. While CD4^+^ single positive thymocytes largely lacked CD122 expression, both classical and alternative Treg cell precursors exhibited increased and almost identical expression levels of CD122 (Figures 1E,F). CD122 expression was even further increased in mature Foxp3^+^CD25^+^ Treg cells (Figures 1E,F).

### Minor Impact of NF-κB for the Development of Alternative CD25^−^Foxp3^+^ Treg Cell Precursors Into Mature CD25^+^Foxp3^+^ Treg Cells

**Figure 2:**
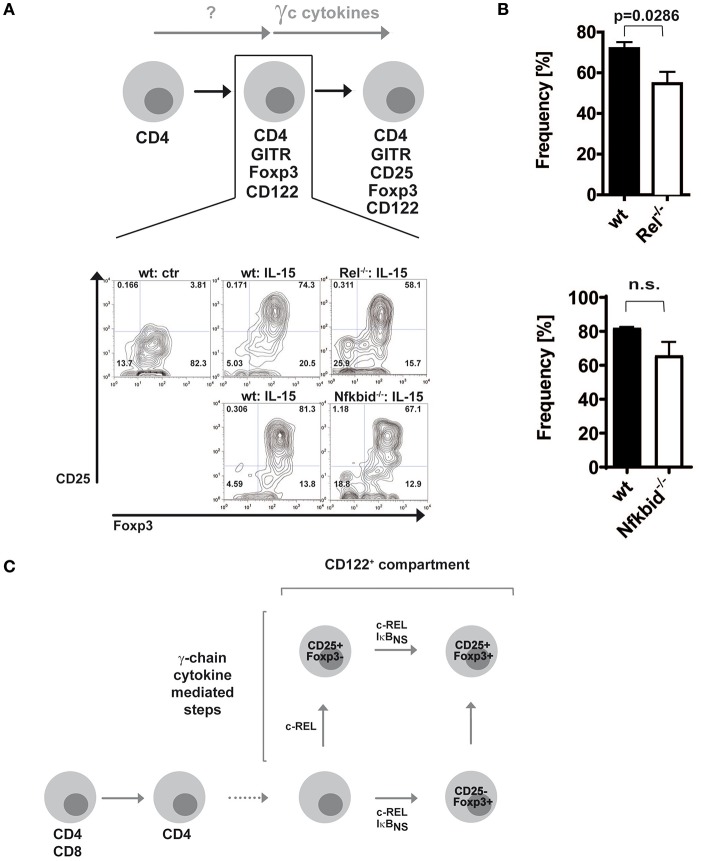
Foxp3^+^CD25^−^ Treg cell precursors differentiate largely independent of NF-κB into mature Treg cells. **(A)** Foxp3^+^CD25^−^ CD122^+^ Treg cell precursors were sorted from wildtype, c-Rel-deficient (*Rel*^−/−^) and IκB_NS_-deficient (*Nfkbid*^−/−^) DEREG mice. Wildtype cells were left untreated or were stimulated with 100 ng/ml IL-15, and IκB_NS_-deficient (*Nfkbid*^−/−^) as well as c-Rel-deficient (*Rel*^−/−^) cells were stimulated with 100 ng/ml IL-15. Representative dot plots are shown. **(B)** Statistical summary to **(A)**. Frequencies of induced Foxp3^+^CD25^+^ cells are shown; open bars indicate c-Rel-deficient (*Rel*^−/−^; upper panel; *n* = 4 both) or IκB_NS_-deficient (*Nfkbid*^−/−^; lower panel; *n* = 3 for wt, *n* = 4 for *Nfkbid*^−/−^) cells, filled bars wildtype. Statistical analyses were performed via two tailed Mann–Whitney tests **(C)** Scheme of contributions of c-Rel and IκB_NS_ for the classical and alternative developmental routes of Treg cells.

Since c-Rel, but not IκB_NS_, regulates the induction of CD25 in the classical route (19), we wondered whether this regulation occurred in the alternative route as well. Thus, we purified alternative Treg cell precursors from wildtype, c-Rel-deficient and IκB_NS_-deficient Foxp3 reporter mice and treated them with IL-15. Indeed, we detected slightly reduced frequencies of CD25^+^Foxp3^+^ Treg cells when c-Rel-deficient alternative Treg cell precursors were stimulated with IL-15 as compared to stimulation of wildtype alternative Treg cell precursors, indicating somewhat impaired CD25 induction (Figures 2A,B). In contrast, IκB_NS_-deficient Treg cell precursors display a normal differentiation into CD25^+^Foxp3^+^ Treg cells. These results indicate that c-Rel regulates Foxp3^+^CD25^−^ alternative Treg cell precursor generation more pronounced than Treg cell maturation, i.e. CD25 induction. Moreover, IκB_NS_ is dispensable for CD25 expression but crucial for Foxp3 induction in both routes of Treg cell development (Figure 2C).

## Discussion

Recent work on NF-κB and Treg cell development has mainly focused on the classical route of Treg cell development. In contrast, it remained unknown whether the generation of CD25^−^Foxp3^+^ alternative Treg cell precursors (8) does also depend on NF-κB. Here, we show that c-Rel-deficient, IκB_NS_-deficient as well as DKO mice display a defect in the generation of alternative Treg cell precursors. So far, the signaling events, which lead to the generation of CD25^−^Foxp3^+^ alternative Treg cell precursors are unknown, but our data indicate that it involves a stimulus, which activates NF-κB and involves the components c-Rel and IκB_NS_. Since TCR stimulation is a prerequisite for Treg cell generation and also a strong stimulus for NF-κB activation, it might be responsible for c-Rel and IκB_NS_ induction.

A second signal required for thymic Treg cell development is through γ_c_ cytokine signaling. However, CD25^−^Foxp3^+^ alternative Treg cell precursors do not express the high-affinity receptor for IL-2 raising the question how these cells respond to γ_c_ cytokines. Our data demonstrates that similar to classical CD25^+^Foxp3^−^ Treg cell precursors also alternative Treg cell precursors express CD122, i.e., the IL-2 receptor β-chain. Downstream of CD122, c-Rel contributes to CD25 induction in Foxp3 expressing cells, i.e., during maturation of alternative Treg cell precursors to mature Treg cells, albeit to a lesser extent than during generation of CD25^+^Foxp3^−^ classical Treg cell precursor (19).

Taken together, our previous data showed that during classical Treg cell development c-Rel regulates CD25 induction in contrast to IκB_NS_ (19). Thus, c-Rel is critical for classical CD25^+^Foxp3^−^ Treg cell precursor generation. In a similar fashion, it contributes to a certain extent to CD25 induction in CD25^−^Foxp3^+^ alternative Treg cell precursors. Finally, IκB_NS_ and c-Rel together control the induction of Foxp3 and thereby alternative CD25^−^Foxp3^+^ Treg cell precursor generation as well as classical CD25^+^Foxp3^−^ Treg cell precursor progression (Figure 2C).

## Data Availability

All datasets generated for this study are included in the manuscript and/or the supplementary files.

## Ethics Statement

All experiments were performed in accordance with regulations according to FELASA, and animals were handled with care and welfare. The protocol was approved by the Niedersächsisches Landesamt für Verbraucherschutz und Lebensmittelsicherheit (LAVES).

## Author Contributions

MS and IS designed the study. MS and CP-S performed the experiments and analyzed the data. AV and JH provided essential reagents and contributed to the study design. IS supervised the whole study. All authors wrote and approved the final version of the manuscript.

### Conflict of Interest Statement

The authors declare that the research was conducted in the absence of any commercial or financial relationships that could be construed as a potential conflict of interest.
